# Professor Lalit Mohan Bariar

**DOI:** 10.1055/s-0045-1808252

**Published:** 2025-05-16

**Authors:** M. Fahud Khurram, Imran Ahmad

**Affiliations:** 1 Department of Plastic Surgery, Jawaharlal Nehru Medical College, Aligarh Muslim University, Aligarh, Uttar Pradesh, India

## Early Life and Education

Born on November 2, 1951, in Bihar, India, Prof. Bariar pursued his medical education with outstanding academic achievements. He completed his MBBS and later his MS (General Surgery) and MCh (Plastic Surgery) at the Institute of Medical Sciences, Banaras Hindu University (BHU), consistently ranking among the top students. His research work and surgical acumen soon set him apart as a leader in the field.

## A Legacy in Plastic Surgery

Prof. Bariar dedicated his career to advancing plastic and reconstructive surgery, particularly in burns management, maxillofacial trauma, and reconstructive microsurgery. He served as the Professor and Chairman of the Department of Plastic Surgery at Jawaharlal Nehru Medical College, Aligarh Muslim University (AMU), where he mentored generations of surgeons, instilling in them the values of precision, compassion, and lifelong learning.

His academic contributions were vast, with numerous research publications in esteemed national and international journals, including the *Indian Journal of Plastic Surgery* , *British Journal of Plastic Surgery* , and *Burns* . His groundbreaking work on hypospadias, cleft lip and palate, and burn wound management continues to shape contemporary plastic surgery practices.

## Academic and Research Contributions

Prof. Bariar was an ardent researcher and a prolific writer. His research on immunological responses in burn injuries, innovative reconstructive techniques, and epidemiological studies on burns and trauma gained widespread recognition. He supervised numerous MS and MCh theses, furthering the body of knowledge in plastic surgery.

He was an active participant in national and international conferences, presenting papers on a wide array of topics, including reconstructive rhinoplasty, electrical burn injuries, and surgical management of maxillofacial anomalies. His role in organizing workshops and hands-on training programs significantly contributed to the skill development of budding plastic surgeons in India.

Prof. Bariar also made significant contributions to academic literature, with notable publications in the *Indian Journal of Plastic Surgery* :

- Masoodi Z, Bariar LM, Haq A. Ellis-van Creveld syndrome with syndactyly, hydrocephalus and cleft palate: a normal variant of Ellis-van Creveld or a new syndrome? (2013). 1
- Tripathy S, Yaseen M, Singh NN, Bariar LM. Interposition arthroplasty in post-traumatic temporomandibular joint ankylosis: a retrospective study (2009). 2
- Khan MM, Yaseen M, Bariar LM, Khan SM. Clinical study of dorsal ulnar artery flap in hand reconstruction (2009). 3
- Bariar LM, Sinha JK, Tripathi FM, Bhattacharya V. Mandibular support following major resections: a brief review (1984). 4
- Bariar LM, Khan MH, Saxena S, Rehman QM. Extensive degloving injury involving left lower limb and penoscrotal skin: a case report (1984). 5
- Bariar LM, Sinha JK, Tripathi FM, Bhattacharya V. Reconstructive rhinoplasty (1982). 6
- Tripathi FM, Khanna S, Khanna NN, Sinha JK, Bhattacharya V, Bariar LM. Marjolin's ulcer (a study of 40 cases) (1982). 7
- Bariar LM, Sinha JK, Tripathi FM, Bhattacharya V. Epidemiological study of hypospadias (1981). 8

## Recognitions and Honors

In recognition of his exceptional contributions, Prof. Bariar held key positions in various medical associations, including the following:

- Life Member, Association of Plastic Surgeons of India (APSI).
- Life Member, National Academy of Burns, India (NABI).
- Life Member, Association of Surgeons of India (ASI).
- President, UP Chapter of APSI.
- Member, Selection Committee, Uttar Pradesh Public Service Commission for Plastic Surgery faculty recruitment.

His dedication to plastic surgery education and training earned him accolades from peers and students alike. His mentorship shaped the careers of many leading plastic surgeons in the country.

In 2008, Prof. Bariar organized the prestigious UPAPSICON, a conference that brought together leading plastic surgeons from across India. The event was a resounding success, attracting esteemed delegates and fostering important academic discussions that furthered the progress of plastic surgery in the country. His dedication to organizing such landmark events showcased his leadership and commitment to professional development in the field.

## Contributions to Burn Care and Reconstruction

He played a pivotal role in advancing burn management protocols in India, advocating for early excision and grafting techniques to improve survival and functional outcomes. His innovative use of biological dressings and amniotic membrane applications helped revolutionize burn wound healing, reducing morbidity and improving long-term recovery.

His work emphasized the need for psychological support for burn survivors, advocating for a holistic approach to recovery that included rehabilitation and social reintegration.

Beyond his clinical work, Prof. Bariar was committed to educating the next generation of surgeons in burn management. He regularly conducted workshops and training programs, sharing his vast knowledge and expertise with young plastic surgeons and general surgeons alike. His contributions to burn prevention campaigns helped raise awareness and reduce the incidence of preventable burn injuries in India.

## A Social and Cultural Icon

Beyond his professional achievements, Prof. Bariar was known for his vibrant social presence. He actively participated in both academic and nonacademic events, making every gathering lively with his enthusiasm and warmth.

An ardent lover of music, Prof. Bariar was also a gifted singer. His melodious voice and deep appreciation for classical and contemporary music made him a cherished presence at social gatherings. He often performed at informal events, delighting friends and colleagues with his soulful renditions. His ability to balance the rigor of academia with the joy of social interactions was a testament to his multifaceted personality.

His sociability and warmth created an environment where students and colleagues felt connected and valued. He believed in building relationships beyond the operating room and classroom, fostering a culture of camaraderie and mentorship that will be remembered for years to come.

## A Lasting Legacy

Even after his retirement, Prof. Bariar remained actively involved in the medical community, contributing to policy discussions, mentoring young surgeons, and advocating for improved health care accessibility. His efforts in burn prevention programs and reconstructive surgery initiatives have had a lasting impact on patient care standards in India.

*“A Life Spent in Service to Others Is a Life Well Lived.”*

—Albert Schweitzer

**Fig. 1:**
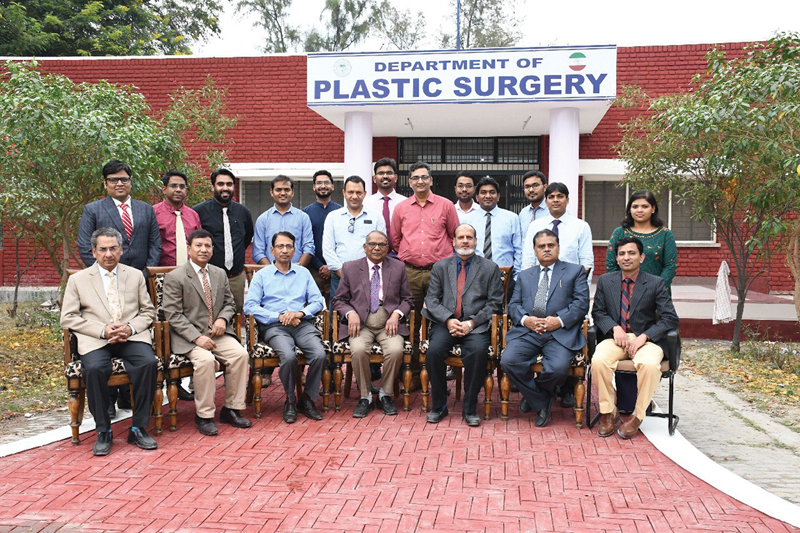
Group photograph: Organizing committee of UPAPSICON 2019.

**Fig. 2:**
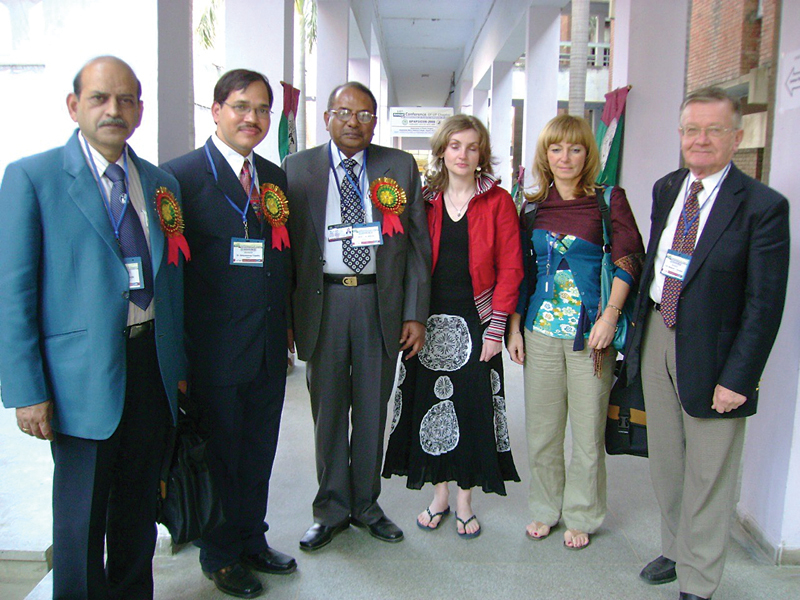
In the year 2008, Prof. L.M. Bariar organized the UPAPSICON at Aligarh. Dr. Pradeep Jain with Dr. S.S. Tripathi (then senior resident) and invited international faculty.

**Fig. 3:**
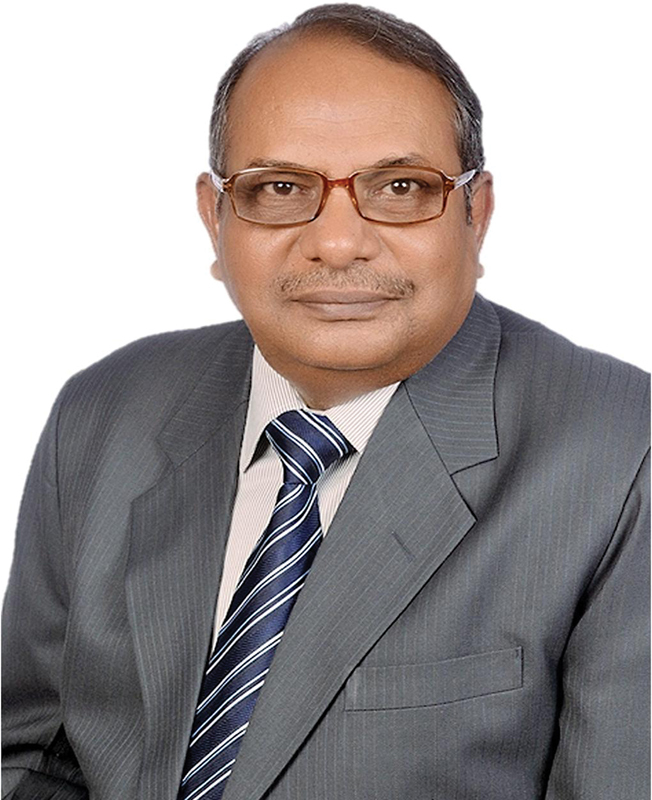
Portrait picture of Prof. L.M. Bariar.

**Fig. 4:**
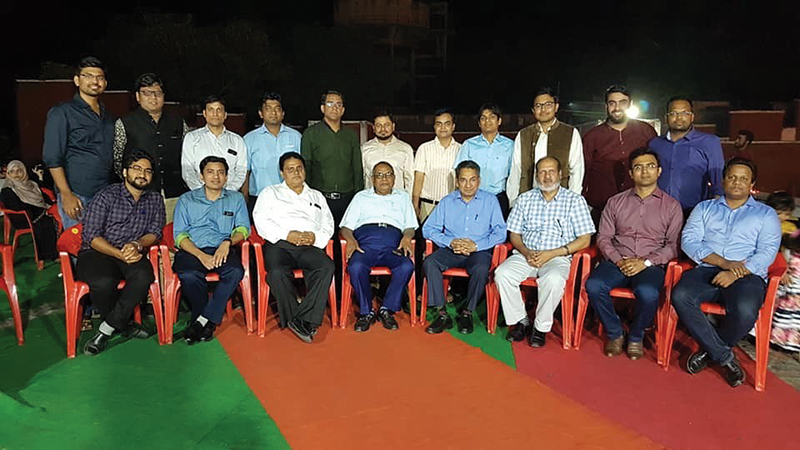
On the occasion of Prof. L.M. Bariar's farewell on December 1, 2016.

**Fig. 5:**
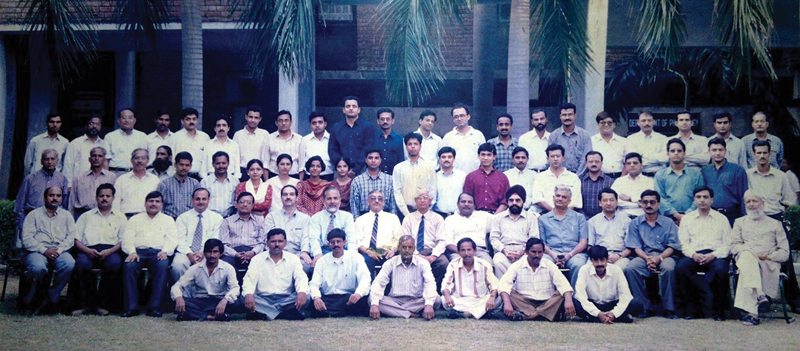
Group photograph: All faculty members and residents of the Department of General Surgery in the year 1999. Plastic surgery was part of the Department of Surgery till 2005.

**Fig. 6:**
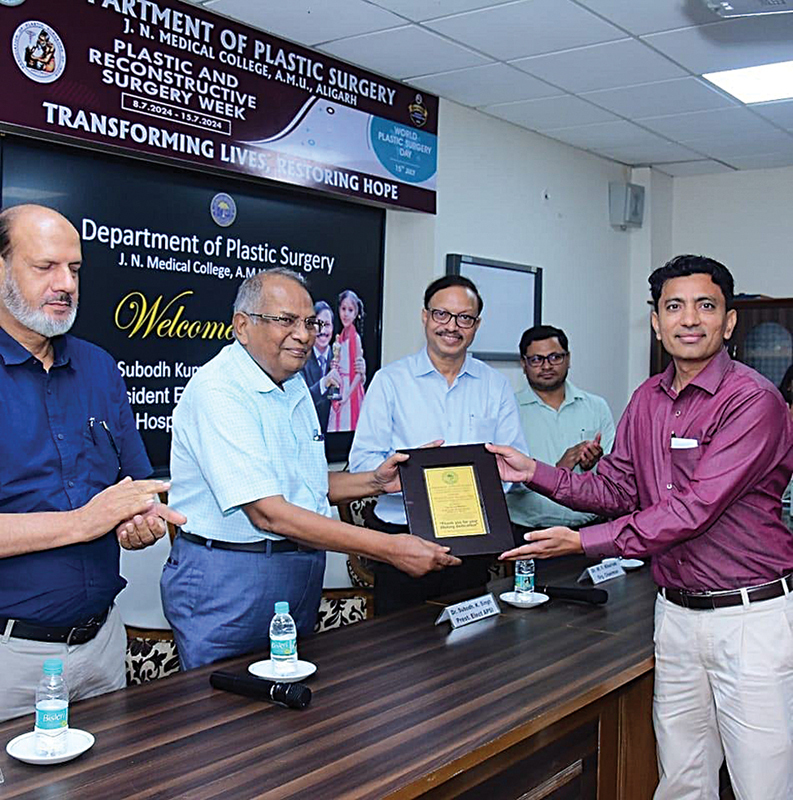
Probably the last function he attended—World Plastic Surgery Day, 2024. He was felicitated with the Great Mentor citation by our current APSI President Dr. Subodh Singh and Head of the Department Dr. M.F. Khurram.

**Fig. 7:**
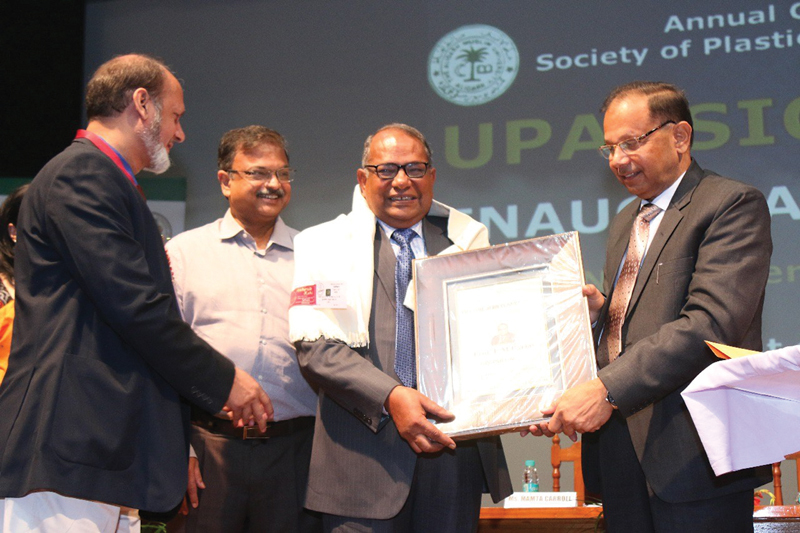
Prof. L.M. Bariar being felicitated with Lifetime Achievement Award during the UPAPSICON 2019 at Aligarh. In the picture are Pro-Vice-Chancellor of AMU, Dean Faculty of Medicine, and Dr. Imran Ahmad.
